# Factors Influencing Disease Prevention and Control Behaviours of Hog Farmers

**DOI:** 10.3390/ani13050787

**Published:** 2023-02-22

**Authors:** Jiamei Wang, Xiangdong Hu

**Affiliations:** Institute of Agricultural Economics and Development, Chinese Academy of Agricultural Sciences, Beijing 100081, China

**Keywords:** hog farms, animal diseases, prevention and control behaviours, African swine fever, influencing factors

## Abstract

**Simple Summary:**

Animal diseases have increasingly become an important external impact that restricts the healthy development of the pig industry. African swine fever has seriously threatened the stable development of the pig industry in China and caused huge economic losses for farmers. The control behaviour by farmers is particularly important in the front line of epidemic prevention. To explore the factors that affect epidemic prevention and control behaviour by farmers, field survey data was empirically analysed. The results show that gender, education level, technical training, breeding years, breeding scale, specialisation, and risk awareness have significant effects on the biosecurity measures taken by farmers.

**Abstract:**

Animal diseases are a serious threat to animal husbandry production and diet health, and effective prevention and control measures need to be explored. This study investigates the factors influencing the adoption of biosecurity prevention and the control behaviours of hog farmers towards African swine fever and provides appropriate recommendations. Using research data from Sichuan, Hubei, Jiangsu, Tianjin, Liaoning, Jilin, and Hebei, we employed a binary logistic model to empirically analyse these factors. Regarding individual farmer characteristics, male farmers emphasised biosecurity prevention and control in farms, with higher education actively influencing the adoption of prevention and control measures. Farmers who received technical training were actively inclined to adopt such behaviours. Furthermore, the longer the duration of farming, the more probable the farmers were to neglect biosecurity prevention and control. However, the bigger and more specialised the farm, the more inclined they were to adopt prevention and control behaviours. With respect to disease prevention and control awareness, the more risk-averse the farmers were, the more they actively adopted epidemic prevention behaviours. As the awareness of epidemic risk increased, the farmers tended to adopt active epidemic prevention behaviours by reporting suspected outbreaks. The following policy recommendations were made: learning about epidemic prevention and improving professional skills; large-scale farming, specialised farming; and timely dissemination of information to raise risk awareness.

## 1. Introduction

The high incidence of and difficulty in controlling major animal diseases has led to a focus on animal health and its relationship with biosecurity [1]. The Food and Agriculture Organization (FAO) considers biosecurity to be directly related to agricultural sustainability, food safety, and environmental protection [2]. The application of biosecurity is the most important measure for avoiding the spread of disease [3]. Disease prevention through biosecurity measures is considered an important factor in improving animal production [4]. In August 2018, China experienced an outbreak of African swine fever (ASF), which spread expeditiously across several provinces, resulting in a steep decline in hog production and dramatic market fluctuations [5]. The outbreak significantly impacted the hog industry, mainly in terms of markets and hog farming entities. The infection led to abnormal fluctuations in the prices of meat, especially pork, disrupting market development and resulting in considerable disparities in regional pork prices [6]. Most farm owners lacked the experience and expertise to prevent and control the virus. Therefore, the measures taken were inadequate, culminating in low-level biosecurity in farming environments. Consequently, the epidemic brought huge economic losses to hog-farming entities [7]. Differences in production size, biosecurity standards, production inputs, and sales practices between hog farms can affect the potential risk of disease transmission and lead to a wide variation in the ability to take preventive measures between farms [8,9]. The current biosecurity situation in China is not promising; the state of some farms is alarming in the wake of the African swine fever, limiting their productivity, and causing heavy economic losses. Therefore, it is necessary to understand the current situation of biosecurity in Chinese hog farms and investigate the factors that influence the prevention and control of epidemics, to improve the level of biosecurity.

Existing studies on the epidemic prevention behaviours of farmers have mainly focused on specific aspects. The epidemic prevention behaviour of farmers is the first step in animal epidemic prevention and control. Farmers usually perform four kinds of epidemic prevention behaviours, which include, the improvement of feeding conditions, the use of vaccines and veterinary drugs, the reporting of animal epidemics, and the treatment of sick and dead livestock [10]. Strengthening biosecurity management measures is the most important means to prevent and control African swine fever in China’s pig farms. The construction of a biosecurity system requires attention to the safety of inputs, optimisation of the feeding environment, management of personnel and vehicles, disinfection, and waste disposal [11]. Zhou et al. [12] examined the disease prevention and control behaviours of farmers and found that small-scale hog farms focused mainly on disinfection, feed and water management, disease prevention and control, and incoming and outgoing vehicle management. Meanwhile, retail farmers emphasised the regulation of the internal and external environment of hog farms, personnel management, and the use of vaccines and drugs. In a study on biocontainment measures in avian influenza prevention and control, Huang et al. [13] focused on preventing rodents and birds, as well as measures to manage incoming vehicles and disinfection of personnel. It has also been suggested that four factors: vaccines, veterinary drugs, disinfection and cleaning, and quarantine, play a crucial role in the prevention and control of hog diseases [14]. The immunisation file, also known as the animal household register, is also an important link in the animal epidemic prevention chain. It records in detail the ages of the animals, entry bar, immunisation date, immunisation identification number, vaccine type, injection, etc [15]. The establishment of immunisation files by farmers is vital to ensure the implementation of compulsory immunisation programs, and the conducting of risk assessments for the epidemic control of animal diseases to accurately grasp the immunisation status and monitor animals, both overall and individually, to avoid missed and late immunisations and to ensure the overall quality of the immunisation [16]. Moreover, ensuring drinking water quality is an important component of livestock rearing and management [17]. Water quality can be evaluated by testing the farm’s drinking water source to check whether the bacterial count exceeds the limit [18]. Since all types of vehicles are in direct contact with the outside world, vehicle biosecurity and management are also important aspects of hog farm biosecurity controls [19].

Scholars have long examined the factors influencing disease prevention and control in farms. He Zhongwei et al. [20] studied the influencing factors of prevention and control behaviours in poultry farmers, taking the individual characteristics, breeding characteristics, epidemic awareness, external environment cognition, and policy implementation as explanatory variables, with which to study the impact of whether farmers adopt epidemic prevention behaviour. Li Yanling et al. [21] analysed the factors affecting epidemic prevention behaviours from four aspects: farmer characteristics, production and operation characteristics, cognitive characteristics, and environmental characteristics. Studying the relationship between epidemic prevention behaviours and the characteristics of poultry farmers, Li Jie et al. [10] used further four prevention behaviours: regular disinfection, whether to believe the official warning, whether to clean the chicken cage, and whether to adhere to the “all-in and all-out” system. Song et al. [22] conducted an empirical analysis of hog farms in the Hubei province and concluded that factors such as farmer literacy, whether they had participated in the training, their knowledge of epidemics, the scale of farming, and whether they had joined a cooperative organisation significantly influenced whether the farmers adopted the disease prevention and control measures. Through a study in the Xinjiang Autonomous Region, Chen et al. [23]. analysed disease prevention and the control behaviours of cattle and sheep breeding cooperatives and found that the education level of the breeders, whether they had received training, their annual income, and the provision of timely assistance were the main influencing factors. Luo et al. [24]. studied the disease prevention and control behaviours in beef cattle and meat sheep farms in the Qinghai province and determined that the education level, whether they had received disease prevention and control training, if the farm maintained records of disease prevention and control, and previous certifications were the main influencing factors determining the adoption of the disease prevention and control behaviours by the farmers. Through literature reviews, it can be summarised that there are four main categories of factors that influence the disease prevention and control behaviours of farmers: the characteristics of the farmer, including their gender, age, and education level; the production and operation characteristics, such as the proportion of the farming income in the total household income; the cognitive characteristics of the farmer, such as their cognitive level, the willingness for disease prevention and control and safety awareness; and environmental characteristics, including socio-economic environment and government policies, and what social services are provided [21,25,26].

While many scholars have adequately researched the disease prevention and control behaviours of farmers alongside any influencing factors, there remain some shortcomings. Most of this relevant research merely focuses on a specific region or province and the representativeness of the samples and the universality of the pattern are, thus, inadequate. For this reason, we used the logistic model to empirically analyse the main factors affecting the relevant animal disease prevention and control behaviours, from the perspective of the farmers. Moreover, we analysed the microscopic research data on hog farmers in Sichuan, Hubei, Jiangsu, Tianjin, Liaoning, Jilin and Hebei, and proposed more targeted countermeasures to improve the prevention and control capacity of the farmers, by providing policy recommendations to improve China’s animal disease prevention and control at the grassroots level.

## 2. Materials and Methods

### 2.1. Data Sources

The data used in this study were obtained from a survey of hog farming and disease prevention and control conducted by the research team in the third and fourth quarters of 2020. The survey area includes seven provinces (municipalities directly under the Central Government) in Sichuan, Hubei, Jiangsu, Tianjin, Liaoning, Jilin, and Hebei. The selection of the provinces and regions for the research mainly used the following approach: each province was ranked from the highest to lowest by the number of slaughtered fattened hogs in 2018, the top 18 provinces whose total production reached 90.78% of the national total were categorised into six grades, with each grade representing a different output level, and one province was selected in each grade as a representative of the corresponding production level. Moreover, considering the geographical variability of hog farming, we selected the provinces to research according to six major geographical sub-regions of China. The north-western region, where a very low number of pigs are produced, was not included in the 18 provinces. The remaining five regions were considered in an integrated/comprehensive manner. When sampling representative provinces in different grades, we selected provinces in different geographical subdivisions, as far as possible, to indicate the variability of the geographical areas used for farming. The specific selections are listed in Table 1.

In addition, some of the provinces selected included those with African swine fever outbreaks, to consider the research content in terms of disease prevention and control. The samples were selected by adopting the principle of stratified stochastic sampling. To gain a comprehensive picture of the occurrence and non-occurrence of the African swine fever outbreak, we selected one infected and one non-infected county per province. Based on the above, we selected the specific locations for research, as shown in Table 2. The survey, which mainly targeted hog farmers, included a combination of field interviews and questionnaires. A total of 267 questionnaires were returned, and 262 valid samples were obtained, excluding those with missing or abnormal data. The items in the questionnaire mainly included basic characteristics of farming entities (age, gender, education level, and years of farming, etc.), the production and operation information of the farm (scale of farming, type of operation, and mode of farming, etc.) alongside the biosecurity prevention and control measures undertaken at the farm, including quarantine, disinfection, as well as vehicle and personnel management.

### 2.2. Descriptive Analysis of the Survey Sample

The survey sample was selected in line with the basic characteristics of hog farming entities, and primarily included males, who accounted for 81.30% of the total sample. Individuals aged between 45–54 accounted for half of the overall group, and those aged 45 or above accounted for 73.66%, in line with the characteristic that the farming entities mainly consisted of middle-aged and elderly male groups. The majority had 6–15 years of education, and their education levels were dominated by junior secondary and senior secondary school, which accounted for 90.08%. The number of years of farming was evenly distributed, with the highest percentage (26.72%) having been engaged in hog farming for 6–10 years. Meanwhile, 79.39% of the farmers had received technical training, whereas approximately 20% had not. The percentage of entities whose farming income accounted for over 50% of their total household income was 92.75%. The proportion of farmers who adopted the farming mode of self-breeding and self-rearing was 69.47%, followed by commercial hog fattening at 26.34%. Additionally, 82.44% of the farms had not participated in industrial organisations, whereas the remaining 17.56% of farms had joined industrial organisations. The basic characteristics of the survey sample distributions are listed in Table 3. The detailed data can be found in the Supplementary Materials File (S1_survey data of pig farms).

Epidemic prevention and control in the farm. According to the code of construction of ecological scale pig farms, the distance of the farm site from the main traffic routes and residential areas should meet certain requirements. The farm boundary should be no less than 300-metres from the main traffic routes, 300-metres from residential areas and other livestock farms, and 1000-metres from areas such as slaughterhouses, livestock and poultry processing plants, livestock and poultry trading markets, waste disposal sites and scenic tourist areas. With respect to the location of farms, the proportion of hog farms located over 300-metres from other farms was 68.70%, those located over 300-metres from residential areas were 69.47%, while 17.94% of the farms were located less than 300-metres from main traffic routes. With the above three indices taken together, the location of more than 60% of the survey sample farms was in accordance with construction regulations. The proportion of farms with an “all-in, all-out” management of feeder hogs is a mere 33.59%, indicating that most farmers buy and sell feeder hogs in separate batches. Furthermore, 90.08% of the farms have separate pens for sows, feeder hogs, and piglets, while 9.92% of farms had not conducted separate zoning management; and 76.72% of the farms had dedicated means of transport. With respect to immunisation records, 87.79% of the farms maintained them. Meanwhile, 83.59% of the farms had separate clean and dirty paths, but 40% of the farms did not conduct effective control of birds, as shown in Table 4. See a Supplementary Materials File (S1_survey data of pig farms) for complete information.

### 2.3. Variable Selection

By reviewing the literature, we found that the disease prevention and control behaviour of hog farmers is mainly influenced by the characteristics of farmers, variables such as farm size and degree of specialisation, and risk awareness of farmers. The variables selected for this study included the following components. The characteristics of the farmers mainly included gender of the farmer, educational level, and whether the farmer had received training in hog farming techniques. The farm characteristics mainly included the number of years of farming, the scale of farming, and the degree of specialisation. The farmers’ risk awareness included their investment risk appetite, whether the hog farms can effectively block the introduction of African swine fever, whether they will report any suspected outbreaks in hog farms, and whether they will report any suspected outbreaks in neighbouring hog farms. The farmers’ disease prevention and control behaviours included, whether the farm has immunisation records, whether water sources were tested, and whether the farm has a dedicated means of transport. The descriptive statistical analysis of the variables is detailed in Table 5. The observed original data of the variables can be found in a Supplementary Materials File (S1_survey data of pig farms).

### 2.4. Model Construction

This study constructed a logistic model to test the factors influencing the adoption of preventive and control behaviours by farmers, which was estimated using the maximum likelihood estimation method. The dependent variables are divided into two categories: y=1 if the hog farmers adopt disease prevention and control behaviours, while y= 0 if not. $X={\left(x_{1},x_{2},\cdots ,x_{k}\right)}^{T}$ denotes the independent variable. The relationship between the probability P(y=1|X) of occurrence of the event and the independent variable was studied, with the logistic regression equation as follows:

(1) $$P(y=1|X)=F\left(\beta_{0}+\beta_{1}x_{1}+\cdots +\beta_{k}x_{k}\right)=e^{\beta_{0}+\beta_{1}x_{1}+\cdots +\beta_{k}x_{k}}/\left(1+e^{\beta_{0}+\beta_{1}x_{1}+\cdots +\beta_{k}x_{k}}\right)+\varepsilon$$

## 3. Results

In this study, Stata software was used to regress a binary logistic model for each of the three factors influencing hog epidemic prevention behaviours. Prior to model regression, variance inflation factor tests were conducted separately to verify the existence of multicollinearity problems. The results showed that the highest value of the inflation factor of the variables did not exceed 2, implying that there was no multicollinearity problem.

The estimation results show that the three models fit well and the LR statistical significance levels are all highly significant at the 1% significance level. As shown in Table 6, in terms of individual characteristics, the gender of the farmer has a significant effect, a 10% significance level, on the adoption of disease prevention and control behaviours, whereas having a dedicated means of transport in place, and its coefficient is significantly negative, indicating that male hog farmers are more inclined to adopt dedicated means of transport. This is related to the fact that male farmers, as the main breadwinners of the family, pay greater attention to taking precautionary measures to ensure that household assets are protected from epidemics. At the 1% level of significance, the number of years of education has a positive effect on the adoption of disease prevention and control behaviours by hog farmers. The higher the educational level, the more active a farmer is in adopting the behaviour of testing the water source. This indicates that people with higher educational levels have greater knowledge and access to information about epidemics and take biological prevention and control at the farm more seriously. Technical training had a positive effect on the building of immunisation records and the disease prevention and control behaviours of dedicated means of transport, at significance levels of 10% and 1%, respectively. This indicates that the farmers’ participation in technical training helped to augment their knowledge of disease prevention and control, improved their relevant awareness, and promoted the relevant behaviours required to prevent epidemics.

With respect to farming characteristics, the number of years of farming has a significantly negative effect on the adoption of disease prevention and control behaviours, with a 1% significance level. This indicates that the more years of farming they possess, the less of a priority the farmers attached to disease prevention and control. When they have adequate farming years, they tend to accumulate extensive farming experience, which, nevertheless, will prompt them to rely on their experience in animal disease prevention and control; thus, they are found to lack in science, which results in gaps in their disease prevention and control behaviours. The size of the farm had a significantly positive effect on the adoption of immunisation records and water testing behaviours at the 5% and 1% levels. This indicates that as the scale of farming increases, farmers pay additional attention to the adoption of disease prevention and control measures to avoid huge economic losses from the onset of diseases. At the 5% significance level, the degree of specialisation contributes positively to both the building of immunisation records and the adoption of dedicated means of transport, suggesting that the higher the proportion of total household income from farming, the greater the focus on the biosecurity situations in the farms, with more human, material, and financial resources invested in farming, which is conducive to the adoption of biosecurity prevention and control behaviours.

With respect to disease prevention and control awareness, the farmers’ appetite for risk had a significant negative effect on building immunisation records and water testing at the 10% and 1% levels, respectively. This suggests that the more risk-averse the farmers were, the more active they were in adopting disease prevention and control behaviours. Conversely, the more risk-seeking the behaviour, the more passive they were in adopting disease prevention and control behaviours. This can be attributed to both types of farmers adopting different attitudes towards risks: active and passive. At the 10% significance level, the more the farmers believed that the farm can effectively intercept the introduction of African swine fever, the more likely they were to adopt active disease prevention and control behaviours and test water sources. In contrast, farmers were more likely to build immunisation records when reporting suspected outbreaks at their farms. At the 1% significant level, farmers were more inclined to have their water sources tested and their means of transport dedicated to the farm when reporting suspected outbreaks found in neighbouring hog farms. These data show that the farmers’ active awareness of disease prevention and control can also generate active disease prevention behaviours and exhibit a positive effect.

## 4. Discussion

Biological vectors and trade are the main methods for African swine fever transmission. Biological vectors generally refer to organisms that infect and carry the African swine fever virus (ASFV). For example, ticks, wild suids, and domestic pigs. Susceptible pigs have direct or indirect contact with these infection sources, which then transmit the African swine fever. In trade, infected pork products, swill, alongside equipment and vehicles are the media of ASFV [27,28]. Epidemiological studies revealed three main routes of ASFV transmission: 46% was spread by unsterilised vehicles and workers, 34% by swill-feeding pigs, and 19% by trans-regional transportation of pigs and products [29]. Unsterilised vehicles and people are the main vectors in the spread of African swine fever, and strict control of vehicles and people entering farms is particularly necessary. The questionnaire survey revealed that most farms have strict control over people and vehicles, whereby foreign vehicles and people are not allowed to enter at will, and farms have special transport as well as vehicle decontamination areas, although this does not cover all farms, and a small number of farms are still loosely managed. In terms of feed and water, only 2.63% of farmers feed their pigs with slop, which is excellent. However, for drinking water, more than half of the farmers have not tested their water sources, which greatly increases the risk of contamination in the farms and poses a threat to the health of the hogs.

Regarding the prevention and control measures of ASF, there is no commercialised vaccine at present, and the focus on prevention and control of ASF is “prevention”. The only control measures are to find infected pigs as soon as possible, to either cull or treat them harmlessly, or block and isolate them [30]. Chai Weihua et al. [31] discussed the measures to strengthen pig breeding management and disease prevention and control based on the three elements of infectious disease control: controlling the source of infection, cutting off the transmission routes, and protecting susceptible populations. Starting with “prevention”, represents cutting off all the paths through which the virus may spread. It is strictly forbidden for people outside the pig farm to enter, and the staff should be disinfected and protected when entering and leaving. Disinfect the materials and vehicles entering the pig farm, and when introducing breeding pigs from outside, it is necessary to set up an isolation and observation period before they become gregarious and attempt to adopt a population management mode of “all-in and all-out” to improve the biosecurity barrier. An Dong et al. [32] concluded that ASF should be prevented and controlled by isolation measures, standardised disinfection procedures for farms, training of farmers on biosecurity knowledge, and international border epidemic prevention. The biosecurity measures taken on the surveyed farms focus on cutting off the means of transmission. Biological control is carried out to prevent birds, rodents, and insects from entering the farm, and to disinfect and control the people and vehicles. In terms of pig management, 90.08% of the farms are zoned, with sows, feeders, and piglets kept separately. However, only 33.59% of the farms adopt “all-in, all-out” management practices for their pigs, which undoubtedly increases the risk for the farms not currently adopting such management practices, as those newly introduced pigs may carry viruses into the farm, increasing the risk of infection for pigs throughout the farm.

In terms of the farmers’ characteristics, men are more aware of epidemic prevention and actively take epidemic prevention actions [33]. As the head of the family, men bear the main business responsibilities, thus, taking active epidemic prevention actions to avoid economic losses. The more educated the farmers are, the more inclined they are to take epidemic prevention measures [34]. The higher the educational background, the more knowledge you will receive, and the higher your ability to acquire information and learn new knowledge. When an epidemic occurs, you will take active defensive measures. Farmers with more years of farming possess rich farming experience, yet this also means that they will follow the old rules. Compared with farmers with fewer farming years, they pay attention to different disease prevention and control measures [35]. The larger the size of the farm when faced with an epidemic, the greater the losses suffered will be, having the intrinsic motivation to adopt epidemic prevention and control behaviours as well as the financial ability to support them [22].

The relationship between risk perception and the adoption of conservation behaviours by farmers has been examined by scholars. The results of Valeeva’s study revealed the direct importance of the intrinsic risk characteristics of farmers for their adoption of risk management strategies [36]. Self-protective (risk-averse) behaviours, in general, directly contribute to the farmers’ decisions to exhibit more specific farm-protective behaviours. This is the exact opposite of the derived empirical results. Conversely, the results of the study indicated that farmers with a higher perception of animal disease risk tend to adopt bird-proofing measures, and those who prefer risk tend to disinfect their vehicles. This is consistent with the results of this paper. The reason for these different results is the different meanings of the measurement indicators. In the questionnaire, “high risk, high return”, “medium risk, medium return” and “low risk, low return” were used as options to measure the farmers’ risk preferences. The empirical results show that farmers with a ‘high risk, high return’ bias are more likely to adopt epidemic prevention behaviours. The outbreak of ASF in China led directly to a shortage of pig stocks and a sharp rise in pork prices [37]. On the one hand, restocking brings huge economic benefits to farmers, but at the same time, they are exposed to the risk of further contracting the ASFV. These farmers, who prefer “high risk, high reward”, are driven to replenish their farms by the huge economic gains, but at the same time take active measures to protect themselves against the risk of the ASFV.

## 5. Conclusions and Recommendations

### 5.1. Conclusions

Most of the farmers in the survey area are middle-aged or older males, mostly above 45 years of age, who have a primary school and junior secondary school education and have received technical training. Most of the farms can implement zoning management, and dedicated means of transport, and have built immunisation records. Nevertheless, there are still approximately 30% of the farms whose layout does not meet the ecological codes of construction, and most of them have not adopted an “all-in, all-out” management style.

With respect to individual farmers’ characteristics, gender, education, and technical training had a significant impact on the adoption of biosecurity measures. Specifically, male farmers paid significant attention to biosecurity control in their farms. The more educated the farmers, the more active they were in adopting biosecurity measures; and farmers who had received technical training were more inclined to adopt biosecurity behaviour than those who had not.

With respect to farming characteristics, the number of years of farming, the scale of farming, and the degree of specialisation had a significant effect on the adoption of disease prevention and control behaviours in the farms. Specifically, the more years of farming, the more likely the farm was to neglect biosecurity control. However, the larger the farm, the greater importance it placed on adopting disease control measures; and the more specialised the farm is, the more inclined it was to adopt disease prevention and control behaviours.

With respect to risk awareness, the more risk-averse a farmer was, the more active they were in adopting disease prevention and control behaviours. Conversely, the more risk-seeking a farmer was, the more passive they were in adopting disease prevention and control behaviours. When farmers have an active awareness of disease prevention and control, they will adopt active disease prevention and control behaviours while reporting suspected epidemics found in neighbouring farms.

### 5.2. Policy Recommendations

#### 5.2.1. Learning about Epidemic Prevention and Improving Professional Skills

Farmers’ knowledge reserves of farming techniques and animal epidemic prevention, their mastery of epidemic prevention measures, and their own scientific literacy, all have a direct impact on the epidemic prevention behaviours adopted by farmers. Therefore, it is important to provide farmers with training in epidemic prevention and control from multiple angles, subjects, and in a variety of ways. Knowledge of epidemic prevention and control should be promoted through the internet, APP, TV, and veterinary medicine dealers. Subjects such as animal husbandry and veterinary departments, scientific research institutes, breeding associations, and government departments should increase technical support for farmers and organise regular epidemic prevention training for farmers to improve their understanding of the principles of various epidemic prevention and control measures and their effectiveness, and to master the know-how of using various prevention and control measures to build the first barrier for epidemic prevention.

#### 5.2.2. Large-Scale Farming, Specialised Farming

Promoting large-scale farming models requires strengthening the promotion and guidance of large-scale farming. Regularly organised visits to large-scale farming demonstration farms for retail and small-scale farmers to raise their awareness of the importance of developing large-scale farming and carrying out biocontainment measures. Improve the degree of specialisation in farming. Promote semi-modern and fully enclosed management. Farms should strictly control the entry and exit of personnel and staff should strictly implement the bathing and disinfection entry system to avoid the risk of virus infection. Environmental disinfection is carried out regularly and quantitatively in pig houses. Establish a sound farm biosecurity prevention and control system to provide a healthier growing environment for pig breeding.

#### 5.2.3. Timely Dissemination of Information, Raising Risk Awareness

Animal husbandry and veterinary departments should release epidemic information in a timely manner. By using new media technology to build a smart platform for animal epidemic prevention and control, timely epidemic information can be released, making it possible for farmers to learn about epidemic information through their mobile phones. Making farmers aware of the serious dangers of epidemics and raising their risk awareness will help increase their willingness to implement epidemic prevention and control measures. The government should vigorously publicise relevant laws and regulations such as the Animal Epidemic Prevention Law and the Code of Practice for the Disposal of Major Animal Diseases to raise the awareness of farming subjects to understand the law and their responsibilities and to know the rules and regulations.

## Figures and Tables

**Table 1:** Number of Slaughtered Fattened Hogs by Province in 2018.

Grade	Province	Number of Slaughtered Fattened Hogs (1000 Head)	Region
**1**	**Sichuan**	**66,383**	**Southwest China**
2	Henan	64,024	South Central China
3	Hunan	59,937	South Central China
4	Shandong	50,823	Eastern China
**5**	**Hubei**	**43,635**	**South Central China**
6	Yunnan	38,505	Southwest China
7	Guangdong	37,574	South Central China
**8**	**Hebei**	**37,096**	**Northern China**
9	Guangxi	34,658	South Central China
10	Jiangxi	31,240	Eastern China
11	Anhui	28,374	Eastern China
**12**	**Jiangsu**	**26,809**	**Eastern China**
**13**	**Liaoning**	**24,958**	**Northeast China**
14	Heilongjiang	19,644	Northeast China
15	Guizhou	18,699	Southwest China
16	Chongqing	17,582	Southwest China
**17**	**Jilin**	**15,704**	**Northeast China**
18	Fujian	14,213	Eastern China

**Table 2:** Regional distribution of the survey sample.

Region	Province (Municipality Directly under the Central Government)	Infected Areas	Non-Infected Areas	Sample Size	Proportion
Northern China	Hebei Province	Baoding (Anguo)	Luannan County, Ningjin County	29	11.07%
	Tianjin	Ninghe District	/	33	12.60%
Northeast China	Jilin Province	Tonghua (Meihekou, Liuhe)	Changchun (Nong’an County, Dehui City)	35	13.36%
	Liaoning	Jinzhou (Linghai)	/	31	11.83%
South Central China	Hubei Province	Huanggang (Tuanfeng)	Huanggang (Xishui)	42	16.03%
Eastern China	Jiangsu Province	Suqian (Muyang)	Yancheng (Funing)	50	19.08%
Southwest China	Sichuan	Chengdu (Jintang)	Bazhong (Siyang)	42	16.03%
Total				262	100%

**Table 3:** Basic characteristics of the survey sample distribution.

Variables	Classification	Sample Size	Proportion (%)	Variables	Classification	Sample Size	Proportion (%)
Gender	Male	213	81.30	Number of years of education	0–5 years	13	4.96
	Female	49	18.70		6–10 years	161	61.45
Age	Under 35 years old	20	7.63		11–15 years	75	28.63
	35–44 years old	49	18.70		16–20 years	13	4.96
	45–54 years	128	48.85	Technical training	Yes	208	79.39
	55–64 years	58	22.14		No	54	20.61
	Over 64 years old	7	2.67	Proportion of farming income	Less than 25%	6	2.29
Years of farming	1–5 years	60	22.90		25%–49%	13	4.96
	6–10 years	70	26.72		50%–74%	46	17.56
	11–15 years	57	21.76		75% and above	197	75.19
	16–20 years	46	17.56	Mode of farming	Self-breeding and self-rearing	182	69.47
	20 years or more	29	11.07				
Level of operation scale	Less than 100 heads	60	22.90		Commercial hog fattening	69	26.34
	100–499 head	81	30.92		Piglet rearing	11	4.20
	500–999 head	35	13.36	Joining an industrial organisation or not	Yes	46	17.56
	1000–4999 heads	76	29.01				
	Over 5000 heads	10	3.82		No	216	82.44

**Table 4:** Prevention and control of internal and external biosecurity in pig farms.

	Variables	Classification	Sample Size	Proportion (%)
External biosecurity				
Site selection	Distance from other farms	(0, 0.3) km	82	31.30
		(0.3, 3) km	113	43.13
		(3, 10) km	53	20.23
		(10, 100) km	14	5.34
	Distance from residential areas	(0, 0.3) km	80	30.53
		(0.3, 3) km	156	59.54
		(3, 10) km	22	8.40
		(10, 35) km	4	1.53
	Distance from main traffic routes	(0, 0.3) km	47	17.94
		(0.3, 3) km	149	56.87
		(3, 10) km	53	20.23
		(10, 20) km	13	4.96
	Existence of natural barriers around the farm (e.g., rivers, mountains, farmland)	Yes	193	73.66
		No	69	26.34
Biological control	Whether birds can be effectively controlled	Yes	157	59.92
		No	105	40.08
	Whether rodents can be effectively controlled	Yes	176	66.17
		No	86	33.83
	Whether insects can be effectively controlled	Yes	163	61.28
		No	99	38.72
Personnel and vehicle management	Can external personnel and vehicles freely enter the farm?	Yes	23	8.65
		No	239	91.35
	Are there vehicles entering the decontamination site?	Yes	219	82.33
		No	43	17.67
	Whether the farm has a dedicated means of transport	Yes	201	76.72
		No	61	23.28
Internal biosecurity				
Cleaning and disinfection	Whether to disinfect the feed?	Yes	207	77.82
		No	55	22.18
	Is there any decontamination operation in the workers’ approach area?	Yes	245	92.11
		No	17	7.89
Feed and drinking water	Whether feed uses kitchen waste (swill)	Yes	7	2.63
		No	255	97.37
	Whether the water source has been tested	Yes	123	46.95
		No	139	53.05
Pig herd management	“All-in, all-out” management of feeder hogs	Yes	88	33.59
		No	174	66.41
	Are sows, feeder hogs, and hoglets managed separately on the farm?	Yes	236	90.08
		No	26	9.92
Infrastructure allocation	Is there a transit site in the inner and outer sites?	Yes	186	69.92
		No	76	30.08
	Separation of clean and dirty paths within the farm	Yes	219	83.59
		No	43	16.41
	Immunisation records	Built	230	87.79
		Not built	32	12.21

**Table 5:** Variable definitions and descriptions.

Variable Name	Meaning of Variables	Mean	Standard Deviation
Explained variable			
Whether the farm has immunisation records	Yes = 1, No = 0	0.88	0.33
Whether the water source is tested	Yes = 1, No = 0	0.47	0.50
Whether the farm has dedicated means of transport	Yes = 1, No = 0	0.77	0.42
Explanatory variables			
Farmer characteristics			
Gender	Male = 0, Female = 1	0.19	0.39
Years of education	Actual number of years of education the farmer has received	9.63	3.08
Training in hog farming techniques	Received = 1, not received = 0	0.79	0.41
Farming characteristics			
Years of farming	Actual number of years the farmer has engaged in farming	12.31	7.43
Farming scale	Less than 100 head = 1, 100–499 head = 2, 500–999 head = 3, 1000–4999 head = 4, more than 5000 head = 5	2.60	1.23
Degree of specialisation	Proportion of farming income in total household income	84.26	21.82
Awareness of disease prevention			
Risk appetite	High risk, high return = 1, medium risk, medium return = 2, low risk, low return = 3	2.42	0.72
Whether the farm is effective in blocking the introduction of African swine fever	Yes = 1, No = 0	0.34	0.47
Whether the farm will report suspected outbreaks	Yes = 1, No = 0	0.66	0.47
Whether the farm will report suspected outbreaks in neighbouring farms	Yes = 1, No = 0	0.60	0.49

**Table 6:** Results of empirical analysis of factors influencing farmers’ disease prevention and control behaviours.

Explanatory Variables	Immunisation Records		Water Source Testing		Whether the Farm Has Dedicated Means of Transport	
	Coefficient	Standard Deviation	Coefficient	Standard Deviation	Coefficient	Standard Deviation
Gender	−0.70	0.46	0.06	0.42	−0.76 *	0.40
Number of years of education	0.14	0.09	0.17 ***	0.06	0.09	0.06
Technical training	0.87 *	0.48	0.67	0.41	0.99 ***	0.39
Years of farming	−0.04	0.03	−0.06 ***	0.02	−0.03	0.02
Farming scale	0.54 **	0.23	0.41 ***	0.14	0.07	0.15
Degree of specialisation	0.02 **	0.01	−0.003	0.01	0.02 **	0.01
Risk appetite	−0.81 *	0.43	−0.63 ***	0.23	−0.34	0.26
Whether the farm is effective in blocking the introduction of African swine fever	−0.86	0.52	0.60*	0.34	−0.20	0.38
Whether the farm will report suspected outbreaks	0.91 *	0.54	−0.08	0.41	0.02	0.41
Whether the farm will report suspected outbreaks in neighbouring farms	−0.19	0.54	1.36 ***	0.40	1.19 ***	0.41
Constants	−0.19	1.83	−1.82	1.21	−1.09	1.30

Note: *, **, and *** denote significance levels of 10%, 5%, and 1%, respectively.

## Data Availability

The data presented in this study are available in the article and supplementary materials.

## Supplementary Materials

The following supporting information can be downloaded at: https://www.mdpi.com/article/10.3390/ani13050787/s1, Excel data file: S1_survey data of pig farms.xlsx.
